# Setu: a pipeline for the robust assembly of SARS-CoV-2 genomes

**DOI:** 10.1128/mra.00237-24

**Published:** 2024-06-07

**Authors:** Nityendra Shukla, Neha Srivastava, Prachi Srivastava, Jitendra Narayan

**Affiliations:** 1 CSIR-Institute of Genomics & Integrative Biology, New Delhi, Delhi, India; 2 Institute of Biotechnology, Amity University, Lucknow, India; DOE Joint Genome Institute, Berkeley, California, USA

**Keywords:** SARS-CoV-2, COVID-19, genome assembly, viral evolution, genome surveillance

## Abstract

Setu is an efficient pipeline integrating currently available open source bioinformatic tools to perform rapid *de novo* assembly to assist tracking of severe acute respiratory syndrome coronavirus 2 genome evolution in clinical data, being particularly useful for institutions with limited computing resources or personnel not familiar with bioinformatic pipelines.

## ANNOUNCEMENT

RNA virus assembly is a challenge (1) due to high error rates during RNA replication, resulting in a high number of mutations and thus exhibiting enormous genetic viral diversity (2). Thus, estimating accurate haplotype reconstruction relies on both robust error correction and read assembly methods (3). The assembly of severe acute respiratory syndrome coronavirus 2 (SARS-CoV-2), a positive-sense single-stranded RNA virus, faces the same challenges. While genome sequencing and bioinformatics have played an important role in the coronavirus disease 2019 pandemic, aiding in viral identification (4), and tracking the transmission and evolution of the virus, a high-quality genome assembly is crucial for effective surveillance and identification of novel lineages.

We present Setu, a pipeline (5) that efficiently performs pre-alignment quality control, using Trimmomatic v.0.39 (6), followed by read selection through read mapping against the SARS-CoV-2 reference using BWA-MEM v.0.7.17 (7). Resulting files are processed using SAMtools v.1.18 (8) to extract mapped reads and BEDTools v.2.30.0 (9) to convert the BAM file into FASTQ. *De novo* assembly is performed using coronaSPAdes v.3.15.5 (10), in addition to reference-assisted scaffolding through Ragout v.2.3 (11), resulting in a single contiguous sequence. Assembly stats are calculated using MetaQUAST v.5.2.0 (12). It is currently optimized for Illumina paired-end sequence data.

Setu was evaluated against *de novo* assembly pipelines MEGAHIT v.1.2.9 (13), ABySS v.2.3.5 (14), IDBA-UD v.1.1.3 (15), as well as against targeted SARS-CoV-2 pipelines TAR-VIR (16) and HAVoC (17) from 125 SARS-CoV-2 paired-end Illumina reads, retrieved from NCBI BioProject PRJNA639066 data set and 79 reads from PRJNA746690 (5). Assembly statistics generated through MetaQUAST v.5.2.0 were used for evaluation. All assemblies were run at k-mer value of 33, where applicable. All evaluations were performed on an HP laptop computer consisting of an Intel Core i5-9300H processor running at 2.4 Ghz consisting of eight threads and 24 GB of RAM to demonstrate Setu’s efficiency.

**Fig 1:**
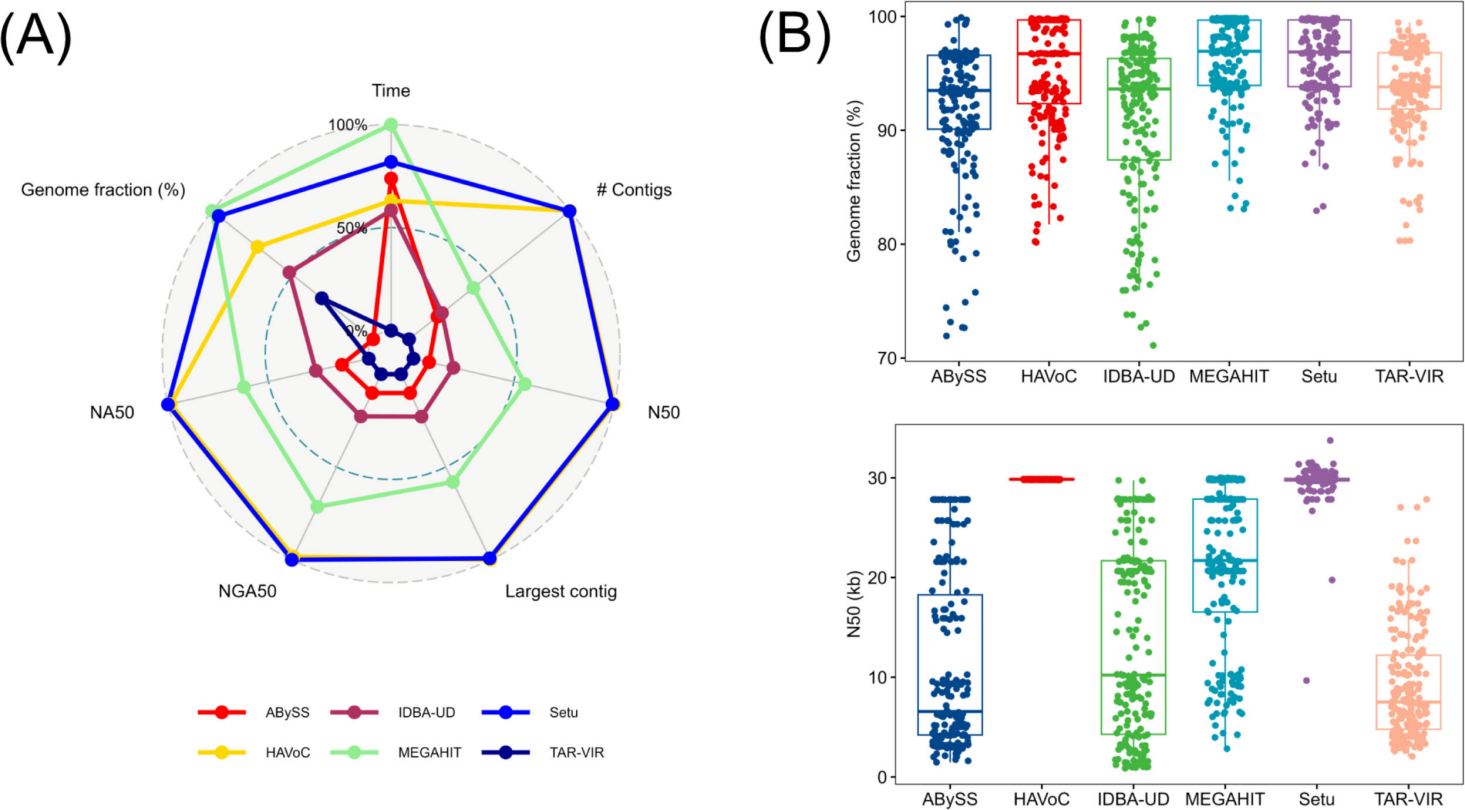
(**A**) Radar plot of evaluation metrics performed (best values at 100%). Setu (blue) had the best performance across all metrices except N50 where HAVoC (gold) performed better. (**B**) Boxplots of genome fraction (above) N50 values (below) across different pipelines.

Setu outperformed all other pipelines (Table 1) (5) in largest contig size, NA50, and NGA50 (Fig. 1A), thus having the highest quality assemblies. It also had the highest mean genome fraction values, covering most of the reference genome (Fig. 1B). HAVoC and Setu had the most contiguous assemblies and joint highest N50 values, respectively (Fig. 1B). MEGAHIT was fastest, completing assembly in 43 minutes, followed by Setu at 70 minutes. None of the pipelines had any extensive memory requirements. It is important to note that out of all pipelines, only Setu and HAVoC perform QC steps before the assembly, while others do not.

**TABLE 1:** Mean statistics of the performance evaluation data set^*a*^

Pipeline	Time (m:s)	# Contigs	Largest contig size (bp)	N50 (bp)	NA50 (bp)	NGA50 (bp)	Genome fraction (%)
ABySS	82:20	6.7	13,019	11,176	11,173	13,017	92.18
HAVoC	98:13	1	29,838	29,838	28,443	28,436	95.21
IDBA-UD	105:32	5.89	15,225	13,814	13,808	15,220	94.37
MEGAHIT	43:52	3.47	21,714	21,123	21,069	23,741	96.22
Setu	70:05	1	29,682	29,669	28,717	28,737	96.40
TAR-VIR	192:11	74.42	11,594	9,066	8,461	11,240	93.52

^*a*^ Statistics here indicate Time; # contigs, total number of contigs; Largest contig size, the size of the largest contig; N50, the longest contig in the genome at 50% assembly length; NA50, the shortest length of aligned bases in the genome at 50% length; Genome fraction, percentage of bases aligned to the reference genome; and the average GC content of each genome assembly.

## Data Availability

The source code, detailed instructions for installation and use are available on GitHub (https://github.com/jnarayan81/setu). We recommend installation of dependencies through the Conda package manager. Setu will remain freely available for the next 10 years alongside instructions for use and any applicable updates. The data used for performance evaluation is publicly through the NCBI Bioproject database at PRJNA639066 and PRJNA746690.
